# Air stimulation in tympanic perforation: inverted nystagmus study

**DOI:** 10.1016/S1808-8694(15)30659-5

**Published:** 2015-10-19

**Authors:** Lucia Kazuko Nishino, Lidio Granato, Carlos Kazuo Taguchi

**Affiliations:** 1 MSc in Health Sciences - Faculdade de Ciências Médicas da Santa Casa de São Paulo, Speech and hearing therapist - Head of the Neurotology Department - Irmandade da Santa Casa de Misericórdia de São Paulo; 2 PhD. Professor - Faculdade de Ciências Médicas da Santa Casa de São Paulo; 3 PhD. Professor - Faculdade de Ciências Médicas da Santa Casa de São Paulo

**Keywords:** air, electronystagmography, tympanic membrane perforation, caloric tests

## Abstract

Response inversion during warm air stimulation is the most controversial finding seen in caloric tests of individuals with tympanic membrane perforation. In such cases, very few studies explore the possible interferences found in the caloric test, bringing about controversies in the interpretation of test results.

**Aim:**

This paper aimed at analyzing warm air stimulation effects in individuals with tympanic membrane perforation in comparison with normal healthy controls.

**Materials and Methods:**

Prospective, non-randomized study in which 48 individuals without vestibular complaints were assessed, 33 had one tympanic membrane perforated and 15 had no ear drum alteration.

**Results:**

39.39% of the individuals had response inversion found during the warm air test. In the absence of this phenomenon, nystagmus responses were symmetrical.

**Conclusion:**

Nystagmus responses to the caloric test in individuals with tympanic membrane perforation were similar to those from healthy controls, with the exception of the fact that they had inverted responses in the warm caloric test.

## INTRODUCTION

The caloric test is considered the most important test within the battery of vestibular tests, because it is capable of providing a topodiagnosis of the vestibular lesion. Much has been done to reduce the intolerability of this procedure, substituting water stimulation for air. This method was first instituted to carry out the test in individuals who could not have water poured in their external acoustic meatus, especially those with tympanic membrane perforations^1^, ^2^, ^3^, ^4^, ^5^.

The air-driven caloric stimulators were developed in order to provide for a safe and reliable test that could be comparable to that with water stimulation. They became very reliable, providing responses much better tolerated for the patients^6^, ^7^, ^8^.

In recent years, many authors^2^,^5^,^9^,^10^ have proposed and studied the systematic use of air-driven stimulation in the caloric test with electronystagmography, replacing the classic water stimulation. This substitution happened for many reasons, among them the possibility of performing the test in individuals with external and middle ear alterations, it is a more practical technique, especially if performed in children, not causing greater discomfort.

However, studies with individuals with middle ear alterations were very little explored^7^,^9^,^11^, ^12^, ^13^, and the investigations which were carried out indicated possible interferences in the caloric test findings, bringing about interpretation controversies to test results.

With the new possibilities brought about by air stimulation, it is necessary to better understand the possible responses to the caloric tests of individuals with tympanic membrane perforations.

The present study aimed at analyzing the air caloric stimulation on individuals with tympanic membrane perforation without otorrhea and without vestibular alterations, in comparison with healthy individuals.

## MATERIALS AND METHODS

Observational case-controlled, non-randomized study approved by the Ethics in Research Committee, under protocol #170/05.

We studied 48 individuals, 15 healthy ones in the control group and 33 with unilateral tympanic membrane perforation, without overt otorrhea and without another concomitant disease in the study group. All the individuals were assessed during 2006 and 2007, in the Speech and Hearing Division of the Otorhinolaryngology Department of our Institution.

Individuals with tympanic perforation were referred to the otorhinolaryngologist, who performed a neurotological evaluation to rule out any peripheral or central vestibular disorder.

All the individuals were explained about the objectives of this study and were invited to participate in it, which started after they agreed to it and signed the informed consent form.

The study participants were submitted to an interview (Attachment 2) to follow inclusion and exclusion criteria, and an otorhinolaryngological evaluation on the day of the vestibular exam, made up of otoscopy, when we characterized perforation extension by percentage of the approximate size of the tympanic membrane perforation and the assurance of no otorrhea.

Basic audiologic evaluation was carried out in an AC-40 two-channel audiometer from Interacoustics, made up of tonal threshold and logo audiometry (speech recognition threshold - SRT and speech recognition index - SRI). The vestibular test (vector-electronystagmography), part of a battery of tests: position nystagmus study, eye movement calibration, open and closed eyes spontaneous nystagmus study, semi-spontaneous nystagmus, saccadic movements, pendular tracking, optokinetic nystagmus, decreasing pendular rotational test and air-driven caloric test (42°C and 18°C) of 80 second duration.

In order to perform the caloric test, the individuals were assessed seating down, with their heads flexed in 60° backwards, with pre-caloric nystagmus to rule out any neck-vestibular interference. During air stimulation, in individuals with tympanic membrane perforation, the test was started in half of the individuals with warm stimulus and on the other half we used cold stimulus. This procedure was carried out in order to check for possible relationship between the discomfort and the stimulus temperature. When we had eventual response inversion in any stimulation, after recording this nystagmus, the heads of these individuals were placed at 180° forward in order to check for vestibular system reactivity.

All the tests were recorded and analyzed by the computer, which performed automatic measures of gain, latency, accuracy and angular velocity of the nystagmus slow component, and also all the necessary calculations in each one of the tests. In the caloric test we considered symmetrical the values below 33% for Labyrinth Preponderance (LP), 22% for Nystagmus Directional Preponderance (NDP) and 30% for cold test symmetry.

The digital computerized vector-nystagmography device we used included, besides the specific software (VECWIN), a light bar to present the visual stimuli. The caloric test stimuli were carried out with the NGR05 otocalorimeter, both from Neurograff Eletromedicina Ind. & Com. Ltda. The normality parameters used were based on the device's software, in other words, 2°/s for absolute hyporeflexia values and 19°/s for hyperreflexia in absolute values, besides the Costa et al. corrections, which consider 24°/s for hyperreflexia values^14^.

The selected subjects were instructed not to have any caffeine laden food or drink alcoholic beverages, 48 hours before the test, and avoid smoking and non-essential medication such as anti-dizziness drugs, anxiolytic and antidepressants, and also to fast for four hours prior to the test. The ones who did not follow these recommendations were re-instructed and rescheduled for testing on another date.

Of the individuals assessed, we excluded those who had sensorineural hearing loss, otorrhea, external acoustic meatus diseases or obstructions, dizziness and the presence of spontaneous and semi-spontaneous nystagmus.

The control group individuals were previously assessed by the otorhinolaryngologist, who ruled out any middle ear alteration; and they were submitted to a complete clinical evaluation.

The data were statistically analyzed by the EPI-INFOâ version 3.3.2 software. The sample was calculated to detect an ODDS RATIO of 1.5. In order to analyze the non-parametric data we used the Fisher's Exact Test and for the parametric data to analyze average and standard deviation values we used the t-Student test. In the entire analyzes we used a significance level below 5%. In the statistically significant results we put an asterisk (*) right after the p-value.

## RESULTS

As far as gender is concerned, the statistical analyses of the control and study groups were homogeneous (Table 1).

**Table 1:** Gender distribution in the control and study groups.

GENDER	control	%	Study	%	TOTAL	%
Male	7	46.67	11	33.33	18	37.50
Female	8	53.33	22	66.67	30	62.50
TOTAL	15	100.00	33	100.00	48	100.00

We did not find statistically significant differences between the groups when we used the Fisher's Exact Test (p=0.2848).

The mean and standard deviation values of the ages of the control group (36.73±11.25) and study group (34.76±10.23) did not show statistically significant differences (p=0.5506) in the t-Student test.

Results from the oculomotor tests and PRPD were all within the normal values established by the software and these were not considered in this experiment.

The sample size was calculated by the odds ratio =1.65; CI95% (0.67-4.04).

Of the 48 individuals studied, 45/48 (93.75%) had symmetrical responses among cold stimuli and three (3/48; 6.25%) had asymmetrical responses, and all of them belonged to the study group, where two individuals had nystagmus responses which were higher in the perforated side and one individual had it in the non-perforated side. The presence of asymmetry in the groups was not significant (p=0.3154), after applying Fisher's Exact Test (Table 2).

**Table 2:** Results from the cold tests in all individuals tested.

COLD TESTS	control	%	study	%	TOTAL	%
Asymmetrical (greater than 30%)	0	0.00	3	9.10	3	6.25
Symmetrical (lower than 30%)	15	100.00	30	90.90	45	93.75
TOTAL	15	100	33	100	48	100

The mean values of the slow component angular velocity (SCAV) results from cold stimuli were studied in the two groups, and they were similar. SCAV mean values in cold stimuli nystagmus did not show statistically significant differences (p=0.5212), based on the t-Student test (Table 3).

**Table 3:** SCAV mean nystagmus values in cold stimulation in all individuals tested.

		SCAV mean values in cold stimuli.		
	N	%	mean	Standard deviation
Control	15	31.25	12.16	6.36
Study	33	68.75	14.28	11.90
Total	48	100.00		

SCAV: slow component angular velocity

In 35 individuals (20 in the study group and 15 in he control group) we studied the four stimulations, and 13 individuals were taken off this analysis, all of them from the study group, (13/33; 39.39%) with response inversion in the warm test. In the group of 35 individuals who underwent the four stimulations we found only one individual with nystagmus directional preponderance greater than 22% towards the perforated side. Alterations present in these four stimulations did not bear statistically significant differences between the groups studied, in the Fisher Exact Test (p=0.5714).

We also studied the mean value of the results from the four stimulations using the Jongkess formula. And we found SCAV mean values of 11.65±5.35 for the control group and of 13.61±7.49 for the study group. The SCAV mean value of the results from all the stimulations in the study group compared to those from the control group did not show differences between the groups in the t-Student test (p=0.3966).

Of the 33 individuals in the study group, 17 (17/33; 51.51%) had tympanic membrane perforation on the left side and 16 (16/33; 48.48%) had it on the right side.

Considering SCAV results in the warm and cold tests, we found 2.60°/s for the minimum value in the warm stimulation and 33.90°/s for the maximum value in the cold stimulation. We did not observe any hyporeflexia value (below or equal to 2°/s), but we found hyperreflexia values (above 24°/s) in the cold tests.

We analyzed the nystagmus responses measuring the slow component angular velocity between the perforated and the intact sides, both in the warm and cold caloric responses. We did not find statistically significant differences in the two sides, they were p=0.3249 for the warm stimulations and p=0.1906 for the cold stimulations in the Fisher Exact Test.

We had hyperreflexia with values above 24°/s in six ears only in the cold tests of the study group. On the perforated side, it happened to two (2/33; 6.06%) individuals and to four (4/33; 12.12%) on the opposite side of the tympanic membrane perforation. The presence of hyperreflexia on the perforated side was not significant on the Fisher Exact Test (p=0.4680)(Table 4).

**Table 4:** Mean sizes of the tympanic perforations associated with the hyperreflexia.

	N	%	Perforation size mean value	Standard deviation
Normoreflexia	31	93.94	50.00	21.87
Hyperreflexia	2	6.06	55.00	21.21
Total	33	100.00		

During air stimulation, eight individuals (8/33;24.24%) reported discomfort during the stimulation of the tympanic membrane perforation ear. Of the 16 individuals who were first submitted to warm stimulation, three (3/16; 18.75%) reported discomfort during warm stimulation and one (1/16; 6.25%) reported it during the cold stimulation. However, with the cold stimulation first, 17 individuals were evaluated, three (3/17; 17.65%) reported discomfort during the cold stimulation and one (1/17; 5.88%) reported it during the warm stimulation.

Thirteen (13/33; 39.39%) individuals from the study group had response inversion during the warm caloric test, which was statistically significant (p=0.0029*) in the Fisher Exact Test.

In all the individuals (n=13) who had response inversion, their heads had been flexed in 120° forward in order to check whether the nystagmus responses came from the labyrinth, and in 100% of the cases there was a change in nystagmus direction, thus proving the reactivity of the peripheral vestibular system.

Regarding the size of the perforation and response inversion, the mean values for both groups were similar, and there was no relation between the perforation size and the presence or absence of response inversion (p=0.6734) in the t-Student test (Table 5).

**Table 5:** Mean sizes of the tympanic membrane perforations associated with the presence or absence of response inversion in the warm caloric test.

Warm caloric test	N	%	Mean sizes of the tympanic perforations	Standard deviation
Without response inversion	20	60.60	49.00	19.44
With response inversion	13	39.40	52.30	25.13
Total	33	100.00		

## DISCUSSION

The caloric test still is the most important one in the battery of vestibular tests^2^,^15^. Numerous publications establish reference values indicating normal values for the air-driven caloric test^14^, ^15^, ^16^, ^17^, ^18^. Although the air-driven caloric test was devised for individuals with tympanic membrane perforation, very few studies have been conducted in these cases^7^,^9^,^11^, ^12^, ^13^,^19^. Most of the studies with air stimulation were carried out in individuals with intact ear drums and proved to be similar to the use of water^3^,^4^,^6^, ^7^, ^8^, ^9^, ^10^,^16^, ^17^, ^18^,^20^. Nonetheless, we still have to present the possibility of a bias when we compare these parameters, since many researchers did their experiments with electronystagmography.

SCAV values of the cold and warm stimulations were calculated by the Neurograff® software and also by the Jongkess formula. In our study we observed that both on the perforation side as well as in the intact side, the SCAV were similar (Table 2, Table 3), and we did not see response differences when a perforation was present, and such data is in agreement with what is presented in the literature^12^.

We have also noticed that the tympanic membrane perforations did not foster the appearance of hyperreflexive responses, even in the presence of large perforations, disagreeing from the results of some authors^13^, who reported that extensive perforations have exaggerated responses. The hyper-reflexive responses found by these authors can be related to the time used in the caloric stimulation, since they used 105 seconds, which can cause nystagmus responses greater that those found in our study in which we used 80 seconds. Other scholars ^9^, used the same time and temperature we did and also found hyperreflexia in 38% of the cases they studied, in disagreement with our findings.

In some cases, caloric stimulation brought discomfort to the patient. In our study, pain or discomfort was reported by 24.24% of the individuals, and this same phenomenon was also observed in individuals ^6^,^20^ without tympanic membrane perforation. On the other hand, there were authors^9^,^11^,^13^,^19^ who did not report this in the cases they studied. Discomfort in the ears with tympanic membrane perforation seem to be associated with the first stimulation, and not the cold or warm temperature, because we noticed it in six of the eight cases who reported a discomfort, in other words, 75% of the individuals with tympanic perforation reported discomfort during the first stimulation. This did not prevent the conclusion of the caloric stimulation, but such fact has to be kept in mind at the time the test is performed.

In our study, individuals with tympanic perforation had nystagmus on the opposite side in a greater frequency to what was expected during the caloric test (39.4%), and this was not in agreement with what is published in the literature^1^,^19^,^21^, ^22^, ^23^ where inverted nystagmus is described as rare. Nonetheless, some authors ^7^,^11^,^13^,^19^ reported that response inversions is more frequently found in perforation cases, very likely due to the presence of otorrhea, without characterizing central involvement. Moreover, our individuals with response inversion did not have any central characteristic, seen during vestibular system assessment in all tests and in the medical interview.

Although not described in the literature, there was great interest in checking whether the tympanic membrane perforation size could facilitate response inversion during caloric stimulation; however, statistically meaningful responses were not found to justify such theory. Response inversion, however, happened both in small and in large perforations (Table 5).

Our attention was also called by the possibility of wrong diagnostic conclusions in cases of response inversion in tympanic membrane perforations, because of the very confusion brought about by the response inversion that happens to most of the professionals who work in this field.

Response inversion in warm stimulation of perforated tympanic membranes happens because of the cold evaporation of occasional moisture in the middle ear mucosa^11^. These authors did a broad study of this phenomenon, describing that the warm air flow in contact with the humid mucosa causes a local cooling effect because of evaporation. This colder temperature is transmitted to the endolymph, causing nystagmus on the opposite side of the expected one. For such phenomenon they used the term secondary nystagmus, which appears after an inverted primary response, better than what is used by other authors^13^,^19^ who defined it as an inverted nystagmus, which can bring wrong inferences since such term is used in the presence of central disorders^1^,^19^,^21^, ^22^, ^23^. Nonetheless, a secondary nystagmus also does not seem to be the best term for such phenomenon, since it can be mistaken with the secondary phase which is the brief nystagmus reaction, usually to the opposite direction, which succeeds, with and without an interval to the post-caloric reaction. Such a definition is also very similar to what happens in tympanic membrane perforation, which we call paradoxical response; however of a different cause which can also be of central origin^24^.

We must also stress that this phenomenon happens on warm stimulation, because in the present investigation we did not find response inversion in any of the cold stimulations.

In our study, the index of response inversion was higher (39.39%; 13/33) than the one found in another study^19^ which reported only 10.57%, and also than those that did not find such effect in any of the cases^9^,^12^.

We did not observe hyporeflexia in absolute values (smaller than or equal to 2°/s) in any of the cases studied; we must stress that the presence of response inversion during the warm air test is not associated with the system's hypofunction, therefore one can not consider or define this labyrinth as deficient, since this would represent an important diagnostic error.

The caloric test with air stimulus was created in order to serve the vestibular diagnosis process of individuals with tympanic membrane perforation or other middle ear diseases; nonetheless we found scarce literature exploring all the characteristics and events that may be found under such conditions. We stress that our study showed execution feasibility on the vestibular assessment of tympanic membrane perforations; however, other associated factors which happened in the many middle ear alterations must be better studied with the aim of clarifying possible influences and minimizing a possible diagnostic error.

Thus, we must also stress that in middle ear alterations, functional or organic variations interfere in different levels of difficulty in controlling and measuring the results of any diagnostic process, in which the caloric test is included.

Further studies must be carried out in order to better understand what middle ear alterations can cause to caloric test results. Physical models must be better investigated, as well as biological models.

## CONCLUSIONS

Cold air stimulation did not caused alteration to the nystagmus SCAV when compared to intact ear drums.

Response inversion in warm air stimulation of perforated tympanic membranes was frequent.

In the absence of response inversions with warm stimulation on post-caloric results, in all the stimuli were similar to those in the control group.
